# Severe abnormal Heart Rate Turbulence Onset is associated with deterioration of liver cirrhosis

**DOI:** 10.1371/journal.pone.0195631

**Published:** 2018-04-10

**Authors:** Christian Jansen, Baravan Al-Kassou, Jennifer Lehmann, Alessandra Pohlmann, Johannes Chang, Michael Praktiknjo, Georg Nickenig, Christian P. Strassburg, Jan W. Schrickel, René Andrié, Markus Linhart, Jonel Trebicka

**Affiliations:** 1 Department of Internal Medicine I, University of Bonn, Bonn, Germany; 2 Department of Internal Medicine II, University of Bonn, Bonn, Germany; 3 Cardiology Department, Hospital Clínic, Barcelona, Spain; 4 European Foundation for the Study of Chronic Liver Failure, Barcelona, Spain; 5 Faculty of Health Sciences, University of Southern Denmark, Odense, Denmark; 6 Institute for Bioengineering of Catalonia, Barcelona, Spain; Medizinische Fakultat der RWTH Aachen, GERMANY

## Abstract

**Background:**

In patients with liver cirrhosis, cardiac dysfunction is frequent and is associated with increased morbidity and mortality. Cardiac dysfunction in cirrhosis seems to be linked to autonomic dysfunction. This study investigates the role of autonomic dysfunction assessed by Heart Rate Turbulence (HRT) analyses in patients with liver cirrhosis.

**Methods and patients:**

Inclusion criteria was (1) diagnosis of cirrhosis by clinical, imaging or biopsy and (2) evaluation by standard 12-lead-ECG and 24h holter monitoring and (3) at least 3 premature ventricular contractions. The exclusion criterion was presence of cardiac diseases, independent of liver cirrhosis. Biochemical parameters were analysed using standard methods. HRT was assessed using Turbulence onset (TO) and slope (TS). The endpoint was deterioration of liver cirrhosis defined as increased MELD and readmission for complications of liver cirrhosis.

**Results:**

Out of 122 cirrhotic patients, 82 patients (63% male) with median Child score of 6 (range 5–12) and median MELD score of 10 (range 6–32) were included. Increasing Child score, INR and decreasing albumin were correlated with TO. In addition, decompensated patients with ascites showed more abnormal TO and TS. During the observation period, patients with more abnormal TO showed significantly higher rate of rising MELD Score at 6 months (p = 0.03). Nevertheless, at least in our collective HRT-parameters were not independent predictors of deterioration of cirrhosis.

**Conclusion:**

Parameters of HRT are closely associated with deterioration of cirrhosis and might be helpful in its prediction.

## Introduction

In patients with liver cirrhosis, autonomic dysfunction (AD) is a frequent finding. Its presence aggravates morbidity and mortality and might play an important role in outcome [1]. AD comprises a broad spectrum of clinical signs and can be assessed by a variety of parameters such as heart rate or blood pressure changes after different maneuvers like Valsalva maneuver or isometric exercise [2]. Heart rate variability (HRV), QT-interval and other parameters have been extensively investigated in liver disease. HRT is a relatively novel HRV measure that describes baroreflex-mediated variations in sinus cycle length after the loss of cardiac output due to a premature ventricular contraction (PVC)[3]. HRT is characterized by two parameters, turbulence onset (TO) and slope (TS), that further describe the intensity of heart rate variation after a PVC. HRT is usually assessed from Holter recordings over at least 24 h[4]. HRT is a useful approach to assess AD[5]. It was initially evaluated for risk stratification in cardiac disease, especially in post-infarct patients. Since then, measures of HRT have been extensively tested in cardiac autonomic dysfunction and have been shown to be associated with prognosis of patients with cardiac diseases [6].

HRT in cirrhosis was described only in one case series of 18 patients and compared to healthy individuals [7]. This series demonstrated that HRT is abnormal in cirrhosis, but its role in the progression of cirrhosis is unclear. Nevertheless the clinical meaning of rate turbulence (HRT) has not been comprehensively investigated in cirrhosis to date.

Our study aimed to evaluate the association of HRT with severity and course of chronic liver disease.

## Patients and methods

### Study design

Inclusion criteria were (1) diagnosis of liver cirrhosis by unequivocal clinical signs, imaging or biopsy and (2) evaluation by 24h-holter monitoring and (3) at least 3 premature ventricular contractions. The exclusion criterion was presence of structural cardiac diseases or cardiac arrhythmias. The composite endpoint was deterioration of liver cirrhosis defined as increased MELD of more than 2 points or readmission in the hospital for complications of liver cirrhosis (e.g. hepatic encephalopathy, newly diagnosed ascites, variceal bleeding, etc.).

### Patients recruitment

122 consecutive cirrhotic patients, who were admitted for treatment of complications of liver cirrhosis at the Department of Internal Medicine I at the University Hospital Bonn, Germany, were screened in this study. Patients with permanent atrial fibrillation (n = 4), artificial cardiac pacemaker (n = 3), structural heart disease (n = 21) or less than 3 premature ventricular contractions (n = 12) were excluded. Finally, 82 patients (52 male) were included in the study and further analyzed. The study was approved by the local Ethics Committee of the University Clinic Bonn (No. 121/14). The patients signed a written inform consent for the procedures in the study. Baseline clinical and biochemical parameters were recorded.

All patients received a 12-lead resting ECG and underwent a comprehensive 2D-transthoracic echocardiography (Philips iE 33 ultrasound system, Amsterdam, The Netherlands). In addition all patients underwent 24h holter ECG monitoring at baseline. For rate correction of QT interval the Bazett formula was applied[8].

The visits of patients were scheduled every 3 months, and the patients were followed for a median of 9 months. During the follow-up, development of CHILD and MELD Score, hepatorenal syndrome, SBP, other infection and death were recorded.

### Holter ECG

All patients underwent 24-h holter monitoring during hospitalization. The holter ECG recordings were archived with the SpiderView TM device (Ela medical Inc., CO, U.S.A.) Mean recording duration was 24 hours. Sample rate was 1000Hz. Standard holter ECG parameters were recorded including number of ventricular premature beat.

### Heart rate turbulence evaluation

All holter tracings were manually reviewed and artifacts were discarded by an operator blinded to the clinical data using the SyneScope^TM^ Version 3.10 software (Ela medical Inc., CO, U.S.A.).

For calculation of heart rate turbulence, only patients that had at least 3 premature ventricular contractions (PVC) were included. The two parameters of HRT, i.e. turbulence onset (TO) and slope (TS), were calculated according to established protocols [9]. Three R-R intervals before and up to 21 intervals after each PVC were analyzed to calculate the sinus cycle length (mean of R_-3_R_-2_ and R_-2_R_-1_), the coupling interval of the PVC, the compensatory pause (first R-R after the PVC) and TO as well as TS. Turbulence onset was defined as the quotient of ((RR_1_ + RR_2_)-(RR_-2_ + RR_-1_)) and (RR_-2_ + RR_-1_) multiplied by 100 (%). Turbulence slope was defined as the maximum positive regression slope assessed over any five consecutive sinus rhythm R-R intervals within the first 15 sinus rhythm R-R intervals after the PVC. To date, there are no established thresholds for differentiation between normal and pathologic values for TO and TS. Therefore, for analysis of association with outcomes, we created two patient cohorts, one with TO values below, one above the TO median of all patients. The analyzes of TO and TS have been shown to be reproducible[9–14], and in our study were performed blinded from the clinical data.

### Statistical analysis

Continuous variables are expressed as the median and range. Non-parametric Wilcoxon and Kruskal-Wallis-Test were used for unpaired comparisons. Correlations were analysed with the Spearman correlation coefficient. Kaplan-Meier curves were used to display deterioration of MELD Score in follow-up as assessed by the Log-rank test. Univariate and Cox regression multivariate analysis (forward step-wise likelihood quotient) was performed to predict decompensation rates. P-values <0.05 were considered statistically significant. Statistical analysis was performed by means of SPSS 22 for Windows (SPSS Inc. Chicago, IL, USA).

## Results

### General characteristics

82 patients were analysed in this study. The median patient age was 61 years (range 19 to 89) and 52 patients were men. The median Child score was 6 (range 5–12), the median MELD was 10 (range 6–32). 20 patients presented large oesophageal varices, 21 patients presented ascites and 36 of the patients were taking beta-blockers. Biochemical parameters were assessed during the study using standard methods and are displayed in Table 1.

**Table 1:** Baseline parameters of the patients (n = 82).

Parameters	Values
**General characteristics**	
gender [female / male]	30 /52
age [years]	61 (19–89)
etiology [alcohol / viral / other]	52 / 20 / 10
Child score	6 (5–12)
MELD score	10 (6–32)
oesophageal varices [absent / small / large ]	44/18/20
ascites [ absent / mild / severe ]	61/13/8
beta-blocker treatment [yes / no]	36/46
**Laboratory values**	
sodium [mmol/L]	140 (115–150)
potassium [mmol/L]	4.15 (2.53–5.83)
serum creatinine [mg/dL]	1.04 (0.6–7.89)
blood urea nitrogen [mg/dL]	38.5 (13–207)
bilirubin [mg/dL]	0.82 (0.18–27.62)
aspartate aminotransferase [U/L]	29 (6–1208)
alkaline phosphatase [U/L]	38 (8–424)
CRP [mg/L]	6.9 (0.4–152)
albumin [g/L]	35 (3.9–50)
INR	1.1 (0.8–2.4)
total white blood cell count [G/L]	5.75 (1.53–25.4)
haemoglobin [g/dL]	11.6 (6.9–16.4)
haematocrit [%]	34 (20–47)
platelet count [G/L]	147 (22–737)
**ECG-Parameters**	
P duration [ms]	90 (50–115)
PQ interval [ms]	135 (80–210)
QRS duration [ms]	100 (70–125)
QTc interval [ms]	430 (321–519)
**Transthoracic echocardiography**	
LV end diastolic volume [mL]	99.8 (41.4–213.7)
LV end systolic volume [mL]	36.15 (12.5–101)
LV ejection fraction [%]	60.35 (45–77.5)

MELD, model for end-stage liver disease; CRP, C-reactive protein; INR, International Normalized Ratio; Hb, Hemoglobin; ECG, electrocardiogram; LV, left ventricular

### 12-channel-resting ECG

The median P duration was 90 ms (range 50–115), the median PQ interval was 135 ms (range 80–210), the median QRS duration was 100 ms (range 70–125) and the median QT interval was 390 ms (range 285–495). 41 patients (50%) had a QTc interval longer than 430 ms (range 321–519). QTc interval did not differ between Child classes. Patients with ascites had a significantly longer QTc interval than those without ascites ((median 469 ms (range 321–519) vs. 426 ms (range 348–515) resp; p = 0.011)). Furthermore, patients with history of bleeding had significant higher QTc intervals than patients without (462 ms (range 389–515) vs. 422 ms (range 321–519), p = 0.001).

### Relationship between HRT, patient characteristics, severity and complications of liver cirrhosis

**Fig 1:**
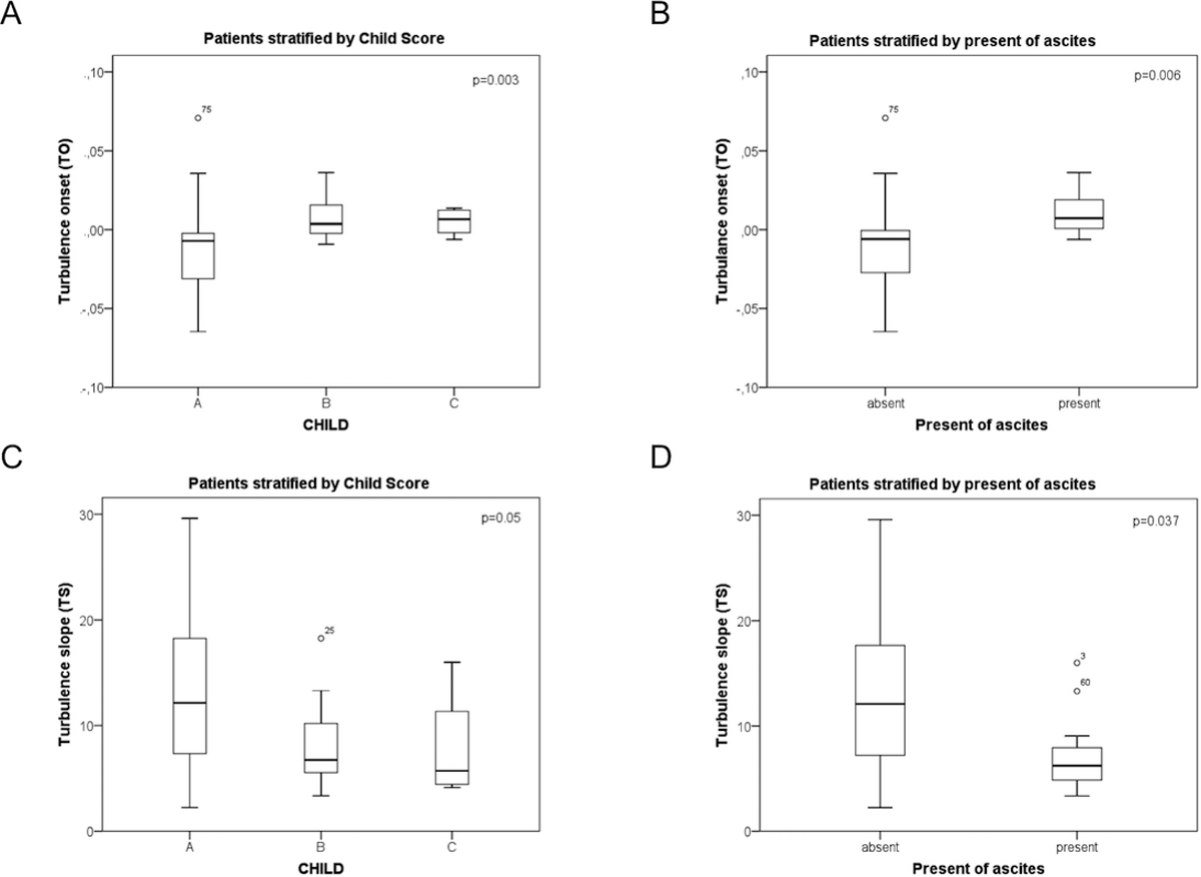
Turbulence onset and turbulence slope differ with respect to the Child score and complications of liver cirrhosis. Turbulence onset was stratified for Child score (A) and presence of ascites (B), as well as turbulence slope (C, D). Data were shown using boxplots and were analysed by Kruskal-Wallis test. Of note, data of two patients lay outside of the shown range, and were not shown to increase readability.

TO correlated significantly with CHILD score (rs = 0.513; p<0.001; Table 2), albumin (rs = -0.343; p = 0.016; Table 2), hemoglobin (rs = -0.395; p = 0.005; Table 2) and haematocrit (rs = -0.435; p = 0.002). Heart rate turbulence parameters TO and TS were significantly deteriorating (i.e. increase of TO and decrease of TS) with increasing severity of liver disease as indicated by increasing Child Class (Fig 1A and 1B). In addition, TO and TS were more pathological in patients with ascites (Fig 1C and 1D). Importantly, no association of HRT parameters was observed with etiology and the intake of non-selective beta-blockers.

**Table 2:** Correlation of heart rate turbulence (TO) with Child-score and biochemical parameters.

	Spearman correlation coefficient	p
Child score	0.513	p<0.001
albumin	-0.343	0.016
hemoglobin	-0.395	0.005
hematocrit	-0.435	0.002

**Fig 2:**
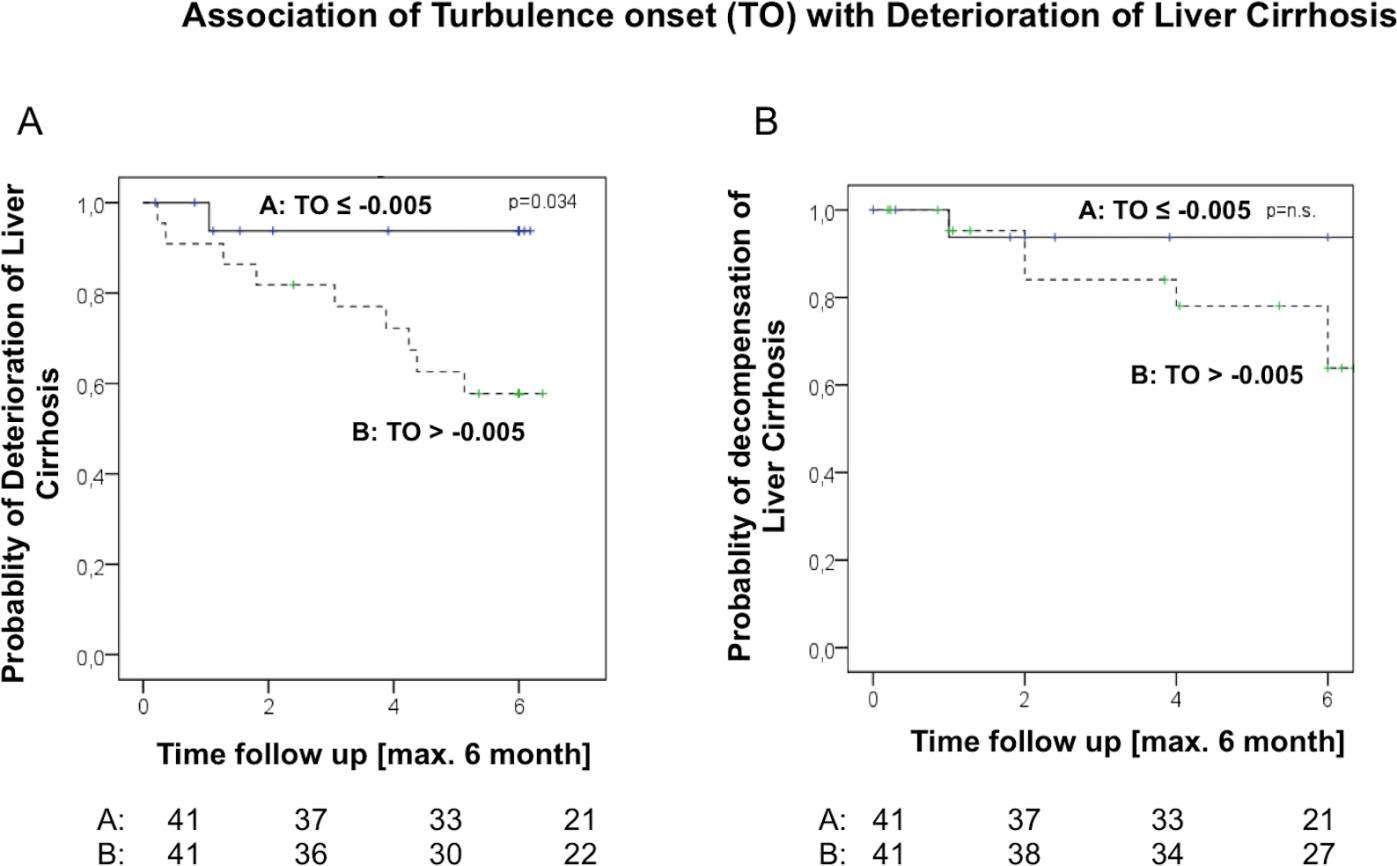
Deterioration of hepatic disease (A) and the decompensation episodes (B) during follow-up stratified using the median of TO-values. Rates are shown using Kaplan-Meier plots and analysed by log-rank test.

When analysing HRT-parameters TO and TS with respect to deterioration of liver disease, severe abnormalities in TO as defined as values above the median TO were associated with higher probability of deterioration of liver disease, as shown by the Kaplan-Meier plot (Fig 2A). The graph demonstrates that more severe abnormalities in TO were significantly associated with deterioration of liver disease (Fig 2A) and showed a trend towards increased number of decompensating events within 6 months (Fig 2B).

Decompensations were developed in 7 patients in the first 6 months, 11 in the first year and 12 in the entire observation period. These were hepatic encephalopathy in 4 patients, newly diagnosed ascites in 2 patients, variceal bleeding in 3 patients, and hepatorenal syndrome in 3 patients. Moreover, we defined a MELD worsening that the baseline increased by more than 2 points. All patients with decompensation showed an increase of the MELD-score of more than 2 points. In total, there were 12 patients in the first 6 months, 23 in the first year and 40 in the entire observation period who met this composite endpoint. The patients who showed an increase of MELD more than 2 points, had at baseline a median MELD of 9 (6–22) and in the follow up a median MELD of 13 (9–31). By contrast, the patients with unchanged or decreasing MELD showed at baseline a median MELD of 13 (7–32) and in the follow up a median MELD of 9 (6–21).

Univariate time-to-event analysis was performed to evaluate the association of TO and other factors with decompensation and deterioration of liver disease after 6 months (Table 3). Besides gender and age, also total white blood count, QRS-duration and TO were significantly associated with decompensation and deterioration of liver disease within 6 months (Table 3). Nevertheless, multivariate cox-regression analysis did not reveal any significantly and independently associated factor with decompensation and deterioration of liver disease at 6 months (data not shown).

**Table 3:** Univariate time-to-event analysis (forward step-wise likelihood quotient) was performed to predict deterioration or decompensation in 6 months.

Deterioration or decompensation in 6 months	p-value	HR	95% confidence interval	
**General characteristics**				
gender	.035	.266	.078	.909
age	.019	1.070	1.011	1.132
**Laboratory values**				
total white blood cell count	.008	1.135	1.034	1.246
**ECG**				
QRS duration	.045	1.063	1.001	1.129
**HRT**				
TO	.032	7.400	4.700	11.600

There was no significant association between any HRV parameter and overall survival (data not shown). This is might be explained by the fact that only 9 patients died during the observation period.

## Discussion

This study demonstrated a relationship between the HRT and deterioration of liver disease in the short-term follow up.

HRV was the first non-invasive method to evaluate autonomic modulation of the sinus node in patients with different cardiac and non-cardiac diseases and to identify patients at risk for an increased cardiac mortality [15] and also has been extensively studied in liver cirrhosis [1, 16–20]. However, the role of HRT, a relatively novel approach to assessment of HRV, has not yet been explored in the outcome of patients with liver cirrhosis. To best of our knowledge, the only description of HRT in cirrhosis, is a small series of 18 patients, that demonstrated that HRT was abnormal when compared to healthy controls [7]. Our study is the first to demonstrate the impact as predictor in patients with liver cirrhosis.

HRV mainly reflects a long-term interaction between neural modulatory mechanism and heart frequency [15]. Briefly, vagal inhibition and sympathetic activation lead to an initial acceleration and thereby alter TO. This probably arises from an inhibition of baroreflex afferent input due to a hemodynamically inefficient ventricular contraction. Then, after the compensatory pause, the increased ventricular filling is responsible for the subsequent deceleration of heart rate [15]. These effects might be involved in the downregulation of myocardial beta-adrenoceptor density, which is has been described in patients with cirrhosis [21, 22]. The present study did not find any association of non-selective beta-blocker (NSBB) intake with HRT parameters, which suggest that HRT might influence beta-receptor density, vice-versa NSBB do not interact with HRT in cirrhotic patients.

The relationship between autonomic dysfunction and development of central hypovolemia is well established in cirrhosis [1]. Especially decompensated patients with ascites show central hypovolemia [1], which might prevent the above mentioned deceleration and therefore impairs HRT. The present study demonstrates the association of HRT parameters with presence of ascites, and substantiates these argumentations. Moreover, low albumin levels induce lower oncotic pressure and also are associated with central hypovolemia[23]. Indeed, there was a clear inverse correlation between albumin levels and TO, which again confirms this pathophysiological concept.

Limitations of this study are that the patients were majorly compensated with a low risk of decompensation and death and therefore lower rate of events, and that the response to stress was not tested to determine cirrhotic cardiomyopathy. The majority of the patients were followed in the out-patient clinic and therefore were rather stable patients. In these patients, HRT was correlated with hemoglobin level, which is a factor determining development of decompensation and acute-on-chronic liver failure in out-patients as recently described[24]. Even though ACLF patients are very severely ill patients, a recently published study demonstrated that these two parameters in stable out-patients determine the development of ACLF [24]. Our study confirms in this relatively compensated cohort of patients, that these two parameters are suitable to identify the patients with deterioration of disease. Moreover, similarly to the acutely decompensated patients in the CANONIC study, white blood count was a factor associated with deterioration of liver disease[25] in our study. These facts underline that the data collected in our study are in line with previously published data. Moreover, our findings support the association of HRT with deterioration of liver disease at least in the short-term follow up. Still our study was unable to show an independent role of HRT in deterioration of liver disease, but offers HRT as a marker associated with deterioration of cirrhosis. It seems that in our study TO seem to be more important than TS, although no pathophysiological explanation can be offered for this finding.

In conclusion, cirrhosis revealed autonomic and cardiac dysfunction and abnormal cardiac distribution of sympathetic activity. Parameters of HRT indicate presence of abnormal sympathetic activity in patients with cirrhosis. Especially TO correlated with the degree and the complications of liver cirrhosis and might be useful to might predict outcome.

## Abbreviations

AD: autonomic dysfunction
CRP: C-reactive protein
Child score: Child-Turcotte-Pugh-score
ECG: electrocardiogram
Hb: Hemoglobin
HE: hepatic encephalopathy
HRS: hepatorenal syndrome
HRT: heart rate turbulence
HRV: heart rate variability
INR: International Normalized Ratio
MELD: Model for End-stage Liver Disease
PVC: premature ventricular contraction
QTc: QTc interval
TO: turbulence onset
TS: turbulence slope
